# Early Literacy and Numeracy Skills in Bilingual Minority Children: Toward a Relative Independence of Linguistic and Numerical Processing

**DOI:** 10.3389/fpsyg.2016.01020

**Published:** 2016-07-07

**Authors:** Paola Bonifacci, Valentina Tobia, Luca Bernabini, Gian Marco Marzocchi

**Affiliations:** ^1^Laboratory Assessment Learning Disabilities, Department of Psychology, University of BolognaBologna, Italy; ^2^Department of Psychology, University of Milano-BicoccaMilan, Italy

**Keywords:** early numeracy, language skills, bilingualism, letter-knowledge, phonemic awareness, Approximate Number System

## Abstract

Many studies have suggested that the concept of “number” is relatively independent from linguistic skills, although an increasing number of studies suggest that language abilities may play a pivotal role in the development of arithmetic skills. The condition of bilingualism can offer a unique perspective into the role of linguistic competence in numerical development. The present study was aimed at evaluating the relationship between language skills and early numeracy through a multilevel investigation in monolingual and bilingual minority children attending preschool. The sample included 156 preschool children. Of these, 77 were bilingual minority children (mean age = 58.27 ± 5.90), and 79 were monolinguals (mean age = 58.45 ± 6.03). The study focused on three levels of analysis: group differences in language and number skills, concurrent linguistic predictors of early numeracy and, finally, profile analysis of linguistic skills in children with impaired vs. adequate numeracy skills. The results showed that, apart from the expected differences in linguistic measures, bilinguals differed from monolinguals in numerical skills with a verbal component, such as semantic knowledge of digits, but they did not differ in a pure non-verbal component such as quantity comparison. The multigroup structural equation model indicated that letter knowledge was a significant predictor of the verbal component of numeracy for both groups. Phonological awareness was a significant predictor of numeracy skills only in the monolingual group. Profile analysis showed that children with a selective weakness in the non-verbal component of numeracy had fully adequate verbal skills. Results from the present study suggest that only some specific components of language competence predict numerical processing, although linguistic proficiency may not be a prerequisite for developing adequate early numeracy skills.

## Introduction

The development of calculation skills is a strong predictor of academic achievement and a key goal of education, but few studies have addressed the determinants of the intuitive development of these skills in preschool years (Purpura et al., 2011), that is, how basic calculation and number skills spontaneously develop in children prior to formal instruction. In the present study, we took into account three main topics in the literature. First, researchers have suggested that children’s mathematical development is primarily determined by an innate approximate number sense (ANS; Dehaene, 1992), which is typically assessed through tasks in which participants are required to discriminate object numerosity (Piazza et al., 2010). In contrast, an increasing number of studies also suggest that language abilities may play a pivotal role in the development of arithmetic skills (e.g., LeFevre et al., 2010; Purpura and Ganley, 2014). Third, the study of calculation skills in the bilingual population (e.g., Mondt et al., 2011; Prior et al., 2015; Rinsveld et al., 2015) has recently received increasing interest with the assumption that bilingualism, and specifically the case of language minority children, may offer a unique opportunity to disentangle the role of language skills in the development of calculation skills.

This assumption is based on the consideration that in many cases bilinguals are less proficient than monolinguals in verbal measures of linguistic proficiency in their L2, and, if numerical processing tested in L2 is highly dependent on linguistic abilities, it follows that bilinguals should underperform compared to monolinguals. Conversely, if numerical skills are primarily based on ANS-related skills, a scarce linguistic proficiency should not necessarily be accompanied by inadequate performance in numerical tasks. An investigation into the linguistic basis of mathematics in language minority children permits a thorough analysis of the relationship between language and mathematics and represents an opportunity to better evaluate individual differences in mathematical development (Vukovic and Lesaux, 2013). However, to our knowledge, few studies so far (Kleemans et al., 2011) have conducted a comparison of bilingual and monolingual children in the preschool years in order to comprehend the relationship between linguistic and numerical knowledge.

### Relationships between Linguistic Skills and Number Knowledge in Monolinguals

According to Von Aster and Shalev’s (2007) four-step model, the development of number acquisition starts from a core-system representation of cardinal magnitude referred to as “Number Sense” (Dehaene, 1997), which provides the basic meaning of numbers. This is a necessary precondition for children to learn to associate a perceived number of objects with verbal labels (Step 2) and Arabic symbols (Step 3). The development of the mental number line (Step 4) is considered to be the fourth and final step, which allows for arithmetic thinking. According to this model, mechanisms of magnitude comparison, language skills and working memory are all prerequisites for an adequate development of calculation skills, although it is suggested that only deficits in the ANS may characterize pure forms of dyscalculia (Von Aster and Shalev, 2007; Piazza et al., 2010; Libertus et al., 2011).

Many studies have suggested that the concept of “number” is relatively independent from linguistic skills (Gelman and Butterworth, 2005; Frank et al., 2008). Nonetheless, it is acknowledged that language plays a role at least in some aspects of numerical cognition; in particular, it seems that verbal processing is a necessary condition for a precise cognitive representation of large numbers (e.g., Dehaene et al., 1999; Spaepen et al., 2011). An increasing amount of evidence is emerging supporting a major role for linguistic skills in arithmetic development (Colomé et al., 2010; Cirino, 2011; Mazzocco et al., 2011; Purpura et al., 2011; Göbel et al., 2014; Purpura and Ganley, 2014). This also seems to be sustained by developmental changes in brain networks underlying numerical processing, with the left angular gyrus supporting the manipulation of numbers in verbal form (Dehaene et al., 2003). Furthermore, studies on clinical populations have documented a high comorbidity of reading and math difficulties (Swanson and Jerman, 2006; Landerl and Moll, 2010; Tobia et al., 2016b), and this has fostered research investigating the role of non-mathematical predictors in mathematical development (Purpura and Ganley, 2014).

Many studies have investigated the role of phonological processing, which is involved in tasks such as number reading (grapheme–phoneme correspondence). If phonological processing is impaired, it may reduce the capacity of working memory (Butterworth, 2005), leading to difficulties in storing and remembering arithmetic facts (e.g., Swanson and Sachse-Lee, 2001; Simmons and Singleton, 2008; Koponen et al., 2013; Vanbinst et al., 2015). There is, however, contrasting evidence regarding the predictive role of phonological skills on mathematical development. For example, in Passolunghi et al. (2007), phonological ability was not found to be a significant predictor in mathematical learning ability in the first year of primary school. In a more recent longitudinal study by Passolunghi et al. (2015), children underwent testing for their phonological skills at the beginning and at the end of the last year of preschool. The results indicated that the children’s levels of phonological awareness that were evaluated at the beginning of the year predicted their numerical abilities that emerged from the assessment at the end of the year. The authors suggested that the influence of phonological skills may be mediated by the age of the children, indicating that it is not constant across development.

One of the other non-mathematical factors that may play a role in mathematical development is lexical amplitude (vocabulary), which is necessary to understand specific language terms (Adams, 2003; Purpura et al., 2011) used in specific instructions, and is highly involved in problem solving (Fuchs et al., 2005). Instruction comprehension and problem solving also involve morphosyntactic comprehension, which has received minor attention in the analysis of the relationship between language and mathematics (Centeno-Cortés and Jiménez Jiménez, 2004). An additional variable that the literature includes among the key predictors of reading development is letter knowledge; because numbers may be considered “special” letters, it might be hypothesized that letter knowledge might as well be a predictor of calculation skills, at least as an indirect index of exposure to print (Caravolas et al., 2005) or as an index of symbolic representation. Finally, there is a broad consensus that both verbal and spatial components of working memory are some of the main predictors of calculation ability. In particular, counting knowledge appears to be more strongly correlated with visuo-spatial working memory than with language precursors (Cirino, 2011). Although some evidence has suggested that the individual components of working memory are related differentially to mathematics (Wilson and Swanson, 2001; Simmons et al., 2012), other results note that the whole working memory system (rather than a specific working memory process) is linked to mathematical knowledge development (Bull et al., 2008; Simmons et al., 2008; Zhang et al., 2014).

Some longitudinal studies are available that consider the predictive role of numerical and non-numerical skills on early calculation abilities in pre-schoolers or in children upon entry into school. As far as numerical skills are concerned, quantity comparison, subitizing, size, and number seriation, counting, and number knowledge have been found to have a predictive role in calculation ability (Göbel et al., 2014; Purpura and Ganley, 2014; Tobia et al., 2016a). Additionally, several linguistic skills are predictive of later calculation skills; this is the case for vocabulary (Purpura and Ganley, 2014), phonological abilities, and verbal IQ (Passolunghi and Lanfranchi, 2012). Finally, some general cognitive factors, such as speed of processing and working memory (Passolunghi and Lanfranchi, 2012; Östergren and Träff, 2013), have a role in predicting early numeracy skills. LeFevre et al. (2010) and Sowinski et al. (2015) tested a set of cognitive precursors of early numerical skills, referred to as the Pathways to Mathematics Model, which, in its latest version (Sowinski et al., 2015), includes three main components – quantitative, linguistic, and working memory – as predictors of numerical (backward counting, arithmetic fluency, calculation, and number system knowledge) and reading variables. It emerged that the quantitative pathway (subitizing, counting, and magnitude comparison) accounted for substantial portions of variance in numerical skills and that the linguistic pathway (vocabulary and phonological awareness) was related to all numerical dependent variables and was also the sole significant predictor of reading.

To summarize, contrasting results have emerged as to the differential role of linguistic competence in calculation ability, and one of the main methodological shortcomings in this line of research is related to the fact that both domains (language and number processing) develop concurrently and with reciprocal interactions in typically developing monolingual children.

### Language and Number Skills in Bilinguals

Some studies have directly addressed the relationship between language and arithmetic skills in adult bilinguals, and, in particular, have analyzed the role of language proficiency and language of training in numerical processing. Among these, Spelke and Tsivkin (2001) highlighted the fact that bilinguals retrieved information about exact numbers more effectively in the instructional language (language of training), whereas they were able to retrieve approximate numbers equally in both of their languages. In secondary school students enrolled in a bilingual program, Saalbach et al. (2013) found a cognitive cost related to language switching from the instructional to the non-instructional language in arithmetic tasks. Similarly, Rinsveld et al. (2015) found that adolescents and young adults were always better at solving complex mathematical tasks in their instructional language; on the other hand, their skills in solving complex calculations in the other language improved with their language proficiency. Another important aspect is the influence of language-specific number word structures in bilinguals’ arithmetic performances (Prior et al., 2015; Rinsveld et al., 2015). These findings suggest that arithmetic processing is sensitive to the linguistic representations of number, and that numerical processing is preferentially processed in the instructional language. This was also shown in an fMRI study (Mondt et al., 2011) on children who learnt mathematics in an instructional language but spoke a different language at home. The authors found that children who performed the task in the instructional language showed a more efficient and specialized pattern of neural activation compared to those performing the same task in their home language. The latter relied more on working memory and visual attentional resources.

Other evidence for the intrinsic relationship between language and mathematics comes from a few studies of bilingual language minority children who were in the course of acquiring their second language (L2) within the scholastic environment. These children can be defined as bilinguals because they speak and are exposed to two or more languages in everyday life (De Lamo White and Jin, 2011). Although bilingualism *per se* does not constitute a risk factor for either linguistic or mathematical skill development, bilingual language minority children often score lower on phonological awareness, vocabulary size, and morphosyntactic competence in their second language during the preschool years (Genesee and Geva, 2006). This offers a new window into the study of the relationship between linguistic competence and number development because, if linguistic competence is a determinant of mathematical skill development, bilingual children may be expected to show lower numerical skills than their monolingual peers.

In summary, if the two domains (linguistic and mathematical) were relatively separate, it would be possible to assume that bilingual children in preschool, although they may have poorer linguistic competences in L2, should not necessarily fall behind monolingual peers in the domain of calculation and mathematical prerequisites. Our research has focused on this aspect, investigating what happens in very young children who have not yet been exposed to formal academic instruction and who learn Italian as their L2. To date, the research that has been conducted with language minority students has focused mainly on their literacy development. An analysis of early numeracy skills in this population and of the relationship with linguistic competence not only provides important theoretical contributions to the connection between language and mathematics but also has implications for assessment procedures and targeted interventions in this understudied population.

## Materials and Methods

### Ethics Statement

The research ethics committee of the University of Bologna approved the LOGOS project, of which the present study is part. Parents of children involved in the study gave informed consent.

### Aims of the Study

To unravel the relationship between language skills and early numeracy in monolingual and bilingual pre-schoolers, the present study focused on three main topics:

(1) Differences between bilingual and monolingual children in linguistic skills and early numeracy abilities. According to the literature review, we expected to find a significant difference in the language domain (Genesee and Geva, 2006). If it emerges that bilingual children underperform in the linguistic domain, and if the linguistic domain is a crucial determinant of numerical skills (Sowinski et al., 2015), these children should also underperform in numeracy skills. On the other hand, if bilinguals show similar numerical skills to monolinguals, this should support the independence of the numeracy domain in relation to the language one.
(2) Linguistic predictors of early numeracy. The second aim of this study was to identify the pattern of concurrent predictors of early numeracy in monolinguals and bilinguals. In particular, numeracy skills with a language component were considered separately from those involving non-verbal numeracy skills, in order to investigate an eventual discrepancy in the pattern of predictors. Among the potential predictors, we considered variables reported by the literature to be significantly linked to numerical abilities in children. We expected at least some linguistic variables to predict the verbal component of numeracy skills in monolinguals. Furthermore, we want to explore whether this pattern is replicated in the bilingual sample. Finally, we expected the non-verbal components of early numeracy to be relatively independent from the linguistic predictors both in monolinguals and bilinguals.
(3) To further investigate the link between linguistic skills and early numeracy, we ran a profile analysis grouping participants based on their profile in numeracy skills, thus identifying children with (1) no difficulties, (2) difficulties only in numerical tasks with a linguistic component, (3) difficulties only in non-verbal number tasks, and (4) difficulties in both components of early numeracy. Verbal skills of these groups were then compared. Profile analysis is an approach that allows for a qualitative understanding of the strengths and weaknesses of the examined population, beyond and above the information derived from group mean scores (Bonifacci and Tobia, 2016). In this study profile analysis was directly aimed at verifying whether a weakness in different components of numerical skills was associated with a deficit in language skills. Considering past studies that found a link between language and some early mathematical skills (e.g., Passolunghi et al., 2015), we expected to find poorer language skills in both monolinguals and bilinguals with difficulties in numerical tasks that have a linguistic component. On the contrary, children with globally adequate skills in early numeracy, or with a selective difficulty in non-verbal numerical tasks, should show unimpaired language abilities, in light of the relative independence of language and non-symbolic numerical skills.

These three methodologies together, applied to a sample of pre-schoolers, offer new and original insight into the relationship between language and number skills before the beginning of formal instruction.

### Participants

A total of 156 children attending 12 public all-day preschool programs in northern Italy took part in this study, which was part of a wider study, the LOGOS Project, aimed at monitoring and reinforcing early indicators of language development and learning difficulties. Of the children, 49.4% were L2 learners of Italian (*n* = 77, 51.9% females; mean age = 58.27 ± 5.90 months, range = 50–77 months), whereas the remaining children were native Italian speakers (*n* = 79, 53.2% females; mean age = 58.45 ± 6.03 months, range = 48–75 months). Children in the two groups were balanced for gender (χ^2^(1) = 0.879, *p* = 0.503) and age (*t*(154) = 0.192, *p* = 0.848). The two groups were recruited from within the same schools and therefore matched for educational exposure, considering, among other aspects, that all the teachers were enrolled in the LOGOS Project, which provides teacher training and pooling of didactic strategies. The schools were in urban and suburban areas that served children from both low-income and middle-income families. This study was carried out in accordance with the recommendations of American Psychological Association (2010), and the research ethics committee of the University of Bologna approved the LOGOS project. Parents of children involved in the study gave informed consent.

Children with Italian as their L2 all spoke minority languages at home. All of these children could be considered early bilinguals because, as specified below, they were all exposed to the Italian language before the age of 3 (Kovelman et al., 2008), as established by consulting their entry into the school system and by collecting information from teachers. The selection criteria were:

(a) exposure to an L1 different from Italian (L2) within the family context;
(b) being born in Italy or arriving in Italy within their first year of life;
(c) having at least 1.5 years of continued exposure to Italian within a school setting;
(d) not being referred to neuropsychiatric units for any range of developmental disorder or sensory or neurological impairment.

The parents of L2 children were mostly from Arabic speaking (20.8%) and Russian-Slavic speaking (22%) countries, but also came from South America (10.4%), Bangladesh (6.5%), Philippines (7.8%), and Romania (10.4%); The remaining participants, 22.1%, originated from other areas (e.g., African French).

For the monolingual group, inclusion criteria were being born in Italy from Italian speaking parents and not being exposed to any other foreign language at home. None of the children had been referred for any range of developmental disorder or sensory or neurological impairment.

The children were attending the second or third year of the Italian preschool program that involves children from 3 to 6 years old. Italian preschool programs do not provide formal instruction in literacy or mathematical skills. However, during the last preschool year, children participate in pre-reading and pre-writing activities, to familiarize them with letters and letter-sound correspondence, and in pre-mathematical games including number songs, classification and seriation of objects, counting and use of worksheets to familiarize with shapes and quantities. These activities tend to occur in the second semester of the last year of preschool, and the children included in the present study were assessed at an earlier age.

### Materials

#### Learning Difficulties Indexes (IDA; Bonifacci et al., 2015)

This assessment battery was developed to evaluate a wide range of linguistic skills in pre-schoolers. It includes six tasks that measure vocabulary, phonological awareness, morpho-syntactic comprehension, phonological memory, story sequencing, and letter knowledge. The internal consistency and reliability values reported refer to the normative sample (*N* = 1416; Bonifacci et al., 2015). The battery is composed of the following subscales:

##### (1) Vocabulary

Children were asked to name 36 images selected for decreasing frequency in spoken language (Barca et al., 2002). The accuracy score, ranging from 0 to 36, was considered. The scale’s Cronbach’s alpha was 0.85. This subtest also allows for an evaluation of speech sound skills, testing 52 main sounds of the Italian language (single phonemes or consonant groups).

##### (2) Phonological Awareness

The battery included four different subtests aimed at assessing phonological awareness: syllable blending (e.g., To-po → Topo; Mouse; six items); syllable segmentation (e.g., Carota → Ca-ro-ta; Carrot; six items); first syllable recognition (e.g., cane-casa; dog-house; four items); and rhymes (e.g., Porta–Torta; Door-Cake; (four items). For the first two tasks, stimuli were presented orally and children were required to provide a verbal answer by blending or segmenting sounds. For the second pairs of tasks, children were presented with a target picture and were required to choose, from among four pictures, which one started or ended with the same sound. Each item received a score of 1 for correct responses and a score of 0 for incorrect answers, for a maximum total score of 20. The scale’s Cronbach’s alpha was 0.84.

##### (3) Morpho-Syntactic comprehension

Children saw three pictures representing three different scenarios and were asked to individuate or manipulate elements of the scenes by comprehending different types of sentences (e.g., active, passive) pronounced by the examiner. For example, the child had to correctly place a picture of a book after hearing the sentence “The book is under the pillow”. Eighteen sentences were presented, and for each of them a score of 2 (correct answer on the first attempt), 1 (correct answer on the second attempt), or 0 (incorrect answer) was assigned. The total score (0–36), was considered. The scale’s Cronbach’s alpha was 0.70.

##### (4) Story sequencing

This task is composed of five pictures depicting a brief tale of a little dinosaur, named Dino, preparing a cake. Each participant was presented with four pictures presented in the wrong order (fixed and predetermined). Image number 1 was given as a prompt, and then the child was asked to arrange pictures in the correct order and to tell the story aloud (this narrative task was not considered in the present study). A score of 1 was given for each picture in the correct order (maximum score: 4). The scale’s Cronbach’s alpha was 0.82.

##### (5) Phonological memory

Children were presented with a non-word repetition task of eight non-words, two 2-syllable, two 3-syllable, two 4-syllable, and two 5-syllable items. Incorrect repetitions were scored 0. Then, a score of 2 was given for perfectly repeated non-words, and a score of 1 was assigned when an articulatory error that had already been detected in the vocabulary task was made. For example, if a child had a difficulty with the phoneme “r” and pronounced the word “rana” (frog) as “lana” in the vocabulary task, a repetition of the non-word “fimedura” as “fimedula” would be scored 1. The total score ranged from 0 to 16, and the scale’s Cronbach’s alpha was 0.72.

##### (6) Letter Knowledge

Children were presented with a picture of a train with one letter (from a to z) in each coach. The experimenter had to choose four letters within the child’s name or first letters of the surname, thus considered to be familiar letters, and four letters chosen randomly but not part of the child’s name, considered to be unfamiliar letters. Then, the experimenter indicated these letters randomly on the train picture, and the child was required to say the sound or the name of the letter. A score of 1 was given for each correct response for a maximum score of 8. The scale’s Cronbach’s alpha was 0.70.

#### Number Sense: Prerequisites (SNUP; Tobia et al., in preparation)

This battery assesses early numeracy skills in pre-schoolers. To be appealing to such young children, each task is presented in a narrative way, and there is a main character, the dragon SNUP, who guides children through the tasks. It is composed of six subtests:

##### (1) Quantity comparison

Children were shown two illustrated baskets and were asked to quickly choose the one with a greater number of fruits in it. The number of fruits varied from 3 to 20, and differences between sets was from 1 to 6 units. A total of 24 items were presented. A score of 1 (correct answer) or 0 (wrong answer) was given for each of them, for a maximum total score of 24. The scale’s Cronbach’s alpha was 0.69.

##### (2) Counting (from 1 to 20)

Children were asked to count 20 buttons dispersed on a board measuring approximately 20 cm × 30 cm. Both knowledge of the verbal sequence of numbers and the acquisition of the biunivocal correspondence principle of counting, namely the ability to link each number word to an individual object, were evaluated. Scores range from 0 to 40, and one point was given for each number word named correctly on the scale of 1–20 and when the child linked one number word to one button. The scale’s Cronbach’s alpha was 0.93.

##### (3) Number line

Children were asked to indicate the point corresponding to 2, 3, 6, 7, and 9 elements (apples placed in a basket) on a 25-cm long line ranging from 0 (empty basket) to 10. The percentage of absolute error (PE; Siegler and Booth, 2004) was calculated. The scale’s Cronbach’s alpha was 0.58.

##### (4) Size seriation, including three tasks

(a) First, children were asked to put four sets of four pictures of the same object (e.g., a house), drawn in different dimensions, in ascending order (seriation with perceptual cues, maximum score: 16); (b) second, a fifth picture for each set was presented, and the child had to put it in the correct place in the ordered composition (insertion, maximum score: 4); (c) finally, children were presented with four series of four pictures of different items (e.g., a bee, a mouse, a cat, and a giraffe), all depicted as the same size, and had to put them in ascending order using their knowledge of the items’ real size (seriation without perceptual cues, maximum score: 16). For each item placed in the correct position, a score of 1 was assigned. The total score ranged from 0 to 36. The Cronbach’s alpha of the size seriation subtest was 0.89.

##### (5) Semantic knowledge of digits

(a) Recognition, (b) reading, and (c) number-quantity association were assessed for digits 1 to 9. The task was organized as a game similar to bingo with numbers. A card containing the digits 1 to 9 randomly distributed on a grid amongst blank squares was used, together with a small bag containing nine number cards, each representing a digit. In the digit recognition subtask, children pointed to the number on the bingo card that had been picked out of the bag and read aloud by the examiner. For the digit reading subtask, children picked a number from the bag and read it aloud. Finally, in the number-quantity association test, children were provided with a card representing sets of elements (baskets of fruit containing from 1 to 9 bananas). The examiner picked and named a digit and children had to choose the set with the corresponding number of elements. For each digit correctly (a) recognized, (b) read, or (c) associated with the corresponding quantity, a score of 1 was given (total score: 0–27). The subtest’s Cronbach’s alpha was 0.93.

##### (6) Visual-spatial memory

Children were asked to remember the position of one to four items (drawings of the dragon SNUP) on 3 × 3 and 4 × 4 grids that were presented for 2 and 4 s, respectively, and then covered. Six 3 × 3 grids containing one, two, or three dragons were presented, and four 4 × 4 grids with three or four dragons on them were shown, for a total of 10 grids. A score of 1 was assigned for each item remembered in the correct position. The maximum total score was 26; Cronbach’s alpha was 0.80.

The Cronbach’s alpha values refer to the normative sample (*N* = 804; Tobia et al., in preparation).

For the administration of both batteries, special attention was given to ascertaining that children correctly understood the instructions: verbal instructions were minimized and examples for each task were provided, in order to make the tasks clear to children. Before starting with the experimental test, participants tried the tasks and feedback was given for both correct and incorrect answers. In this phase, if a child showed difficulties understanding the task, the instructions and an example were repeated.

### Data Analysis

All the raw scores were converted into scaled scores (Mean = 10, *SD* = 3), according to the batteries’ norms. Differences between L2 and native Italian speakers in the linguistic and mathematical prerequisites of learning were analyzed with two multivariate analyses of variance (MANOVA), one including the subscales from the IDA battery, and one including the ones from the SNUP battery.

**FIGURE 1:**
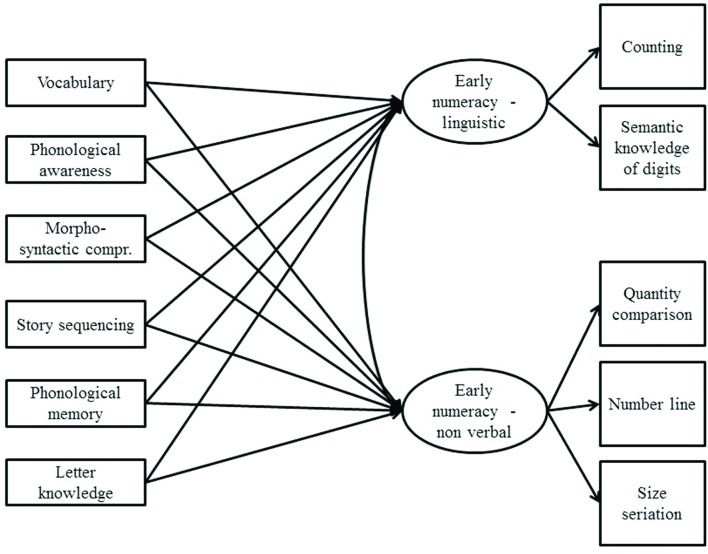
**Model tested with structural equation modeling**.

As a second analysis, a multigroup structural equation model (SEM; e.g., Kline, 2010) including a confirmatory factor analysis (CFA) and a path analysis was applied using MPlus (Muthén and Muthén, 1998–2010). The CFA identified two latent variables within the SNUP battery. The first one included the early numeracy tasks that had a linguistic component (i.e., Counting and Semantic knowledge of digits), whereas the second factor included early numeracy variables without a linguistic component (i.e., Quantity comparison, Number line, and Size seriation). Visual-spatial memory was not considered because it is a prerequisite of mathematical skills, but it is not an effective component of number sense as it is a general cognitive precursor. A path analysis was used to examine the relationship between these dependent variables and the linguistic tasks included in the IDA battery that were considered as potential predictors. The model was tested on monolinguals and bilinguals with the multigroup technique. The complete model analyzed is presented in **Figure 1**.

Multiple indices were used to evaluate model fit, including Chi-square, Root Mean Square Error of Approximation (RMSEA), Comparative Fit Index (CFI), Tuker-Lewis Index (TLI), and Standardized Root Mean Squared Residual (SRMR). A non-significant Chi-square, an RMSEA result no greater than 0.06, CFI and TLI results of 0.95 or better, and an SRMR result of less than 0.08 suggested good model fit for the ML estimator used for this analysis (Hu and Bentler, 1999).

Finally, profile analysis in early numeracy was performed. Children were classified as follows:

(1) no difficulties in early numeracy: scaled scores on all the SNUP tasks > 6;
(2) difficulties only in the early numeracy tasks with a linguistic component: a scaled score ≤ 6 in Counting and/or in Semantic knowledge of digits;
(3) difficulties only in the non-verbal early numeracy tasks: a scaled score ≤ 6 in one or more tasks among Quantity comparison, Number line, and Size seriation;
(4) difficulties in both the components of early numeracy: scaled scores ≤ 6 in at least one task between Counting and Semantic knowledge of digits, and in at least one task among Quantity comparison, Number line, and Size seriation.

The cut-off of a scaled score of 6 was chosen because it corresponds to a *z* score of about -1,3 SD, which is usually adopted as a criterion for identifying at risk performances. Then, a chi-square test for independence was performed to analyze the links between the categories of difficulties in early numeracy and the monolingual or bilingual condition. Finally, a MANOVA investigating the effect of the different types of profiles of early numeracy on the performance to the IDA tasks was performed. Tukey *post hoc* tests were run to analyze differences between the four profiles. All of the analyses, with the exception of the SEM, were conducted using SPSS 21 (SPSS Chicago, IL USA).

## Results

### Differences in the Prerequisites of Learning

The MANOVA revealed a significant multivariate effect for Group (Pillai’s Trace = 0.313, *F*(6,144) = 10.920, *p* < 0.001, η^2^ = 0.313) on the set of subtests included in the IDA battery. Scaled scores obtained by the children and the results of the univariate analyses are presented in **Table 1**.

**Table 1:** Descriptive statistics and results of the univariate analysis for monolinguals and bilinguals on the “Learning Difficulties Indexes” (IDA) and “Number Sense: Prerequisites” (SNUP) batteries.

		Monolinguals mean (SD)	Bilinguals mean (SD)	*F*(1,150)	*P*	η^2^
IDA	Vocabulary	10.55 (2.43)	7.51 (2.33)	61.72	<0.01	0.293
	Phonological Awareness	9.95 (2.89)	8.55 (2.68)	9.53	0.002	0.060
	Morpho-syntactic comprehension	10.39 (2.72)	8.11 (2.32)	30.95	<0.01	0.172
	Story sequencing	9.70 (2.24)	9.08 (2.41)	2.65	NS	0.018
	Phonological memory	8.78 (2.73)	7.69 (2.59)	6.24	<0.05	0.040
	Letter knowledge	9.30 (2.35)	8.17 (2.20)	9.31	<0.01	0.059
SNUP	Quantity comparison	10.36 (2.99)	9.74 (2.89)	1.670	NS	0.011
	Counting	9.35 (3.12)	8.82 (3.12)	1.062	NS	0.007
	Number line	13.41 (3.16)	12.27 (3.08)	4.991	<0.05	0.032
	Size seriation	10.72 (3.20)	9.14 (3.08)	9.525	<0.01	0.060
	Semantic knowledge of digits	10.43 (2.77)	9.41 (2.79)	5.120	<0.05	0.033
	Visual-spatial memory	9.77 (2.80)	10.52 (2.68)	2.837	NS	0.019

In **Table 1**, descriptive results of the children’s performance on the SNUP battery are also reported. A significant multivariate effect of Group was also found for early numeracy skills (Pillai’s Trace = 0.127, *F*(6,144) = 3.479, *p* = 0.003, η^2^ = 0.127). However, as shown in **Table 1**, the results of only half of the early numeracy tasks significantly differed between the two groups.

### Language Predictors of Early Numeracy

All the fit indices suggested that the multigroup SEM fit the data well: χ^2^(50) = 50.993, *p* > 0.05; RMSEA = 0.016 (90% confidence interval = 0.000–0.076); CFI = 0.995; TLI = 0.992; SRMR = 0.061.

**FIGURE 2:**
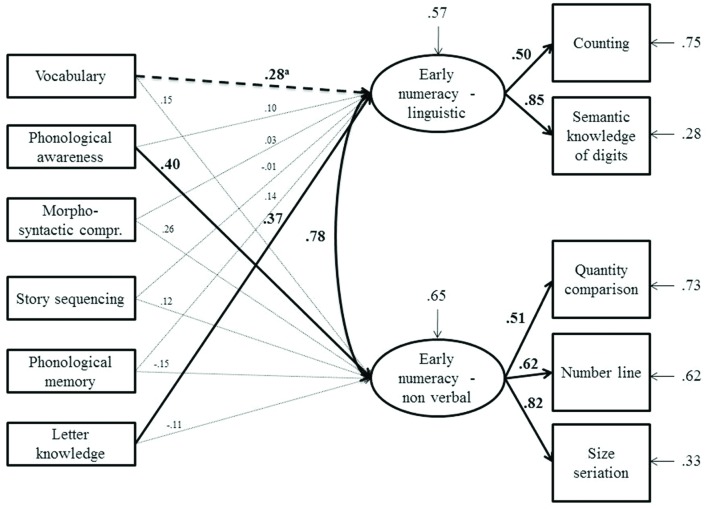
**Model tested with structural equation modeling on monolinguals**. Bold arrows and coefficients represent significant relationships at *p* < 0.01; ^a^*p* = 0.068. The arrows above the latent variables represent the residual variance for the dependent variables.

**FIGURE 3:**
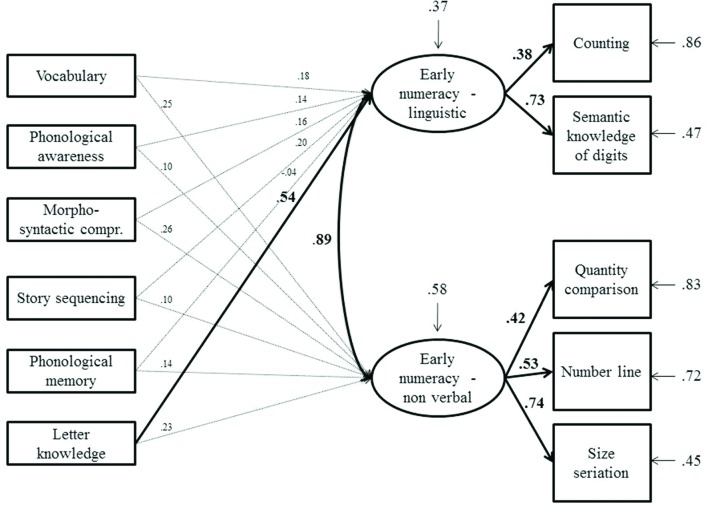
**Model tested with structural equation modeling on bilinguals.** Bold arrows and coefficients represent significant relationships at *p* < 0.01. The arrows above the latent variables represent the residual variance for the dependent variables.

**Figures 2** and **3** describe the model fitted to the data obtained from the monolingual and bilingual groups, respectively. The CFA’s results were similar in the two groups, showing that the two latent variables corresponding to linguistic and non-verbal components of early numeracy were consistent across groups. On the contrary, the path analyses revealed a different pattern of predictors. As far as monolingual children were concerned, the linguistic component of numeracy was predicted by letter knowledge and, marginally, by vocabulary. The non-verbal component was predicted by the phonological awareness task.

In bilinguals, as in monolinguals, letter knowledge predicted the linguistic component of early numeracy. On the contrary, none of the linguistic variables considered predicted the non-verbal component.

### Profiles of Early Numeracy Difficulties

The number of monolingual and bilingual children in each of the four early numeracy profiles, derived by the classification of early numeracy difficulties, is reported in **Table 2**.

**Table 2:** Number of monolingual and bilingual children included in the four categories describing early numeracy difficulties.

		Monolinguals	Bilinguals
Profiles based on performance on the SNUP tasks	(1) No difficulties	48 (60.8%)	33 (42.9%)
	(2) Difficulties only in tasks with a linguistic component	14 (17.7%)	16 (20.8%)
	(3) Difficulties only in non-verbal tasks	9 (11.4%)	16 (20.8%)
	(4) Difficulties in both the components of early numeracy	8 (10.1%)	12 (15.5%)

The chi square test showed that there was independence between profiles of early numeracy abilities and being monolingual or bilingual (χ^2^(3) = 5.646, *p* = 0.130). Considering this result, a MANOVA was run on the entire sample.

A MANOVA was performed to analyze the main effect of Profile on early numeracy on the language tasks included in the IDA battery. The results showed a significant multivariate effect of the children’s Profile (Pillai’s Trace = 0.327, *F*(18,432) = 2.934, *p* < 0.001, η^2^ = 0.109). Descriptive statistics, results of the univariate analysis and *post hoc* tests are presented in **Table 3**.

**Table 3:** Profile analysis.

		Vocabulary	Phonological awareness	Morpho-syntactic comprehension	Story sequencing	Phonological memory	Letter knowledge
Profiles based on the performance at the SNUP tasks	(1) No difficulties	9.87 (2.74)	10.24 (2.90)	10.29 (2.67)	9.99 (2.14)	9.01 (2.52)	9.38 (2.34)
	(2) Difficulties only in tasks with a linguistic component	8.47 (2.94)	7.93 (2.50)	8.27 (2.68)	8.43 (2.19)	6.80 (2.80)	7.83 (1.93)
	(3) Difficulties only in non verbal tasks	8.48 (2.60)	9.40 (2.35)	8.56 (2.22)	8.76 (2.54)	8.52 (2.62)	8.80 (2.25)
	(4) Difficulties in both the components of early numeracy	7.45 (2.28)	7.30 (2.13)	7.70 (2.49)	9.35 (2.50)	7.10 (2.29)	7.60 (2.23)
MANOVA	*F*(3,150)	5.487	9.642	8.572	4.171	6.879	5.454
	*P*	≤0.001	≤0.001	≤0.001	≤0.01	≤0.001	≤0.001
	η^2^	0.101	0.164	0.149	0.078	0.123	0.100
	*Post hoc*	4 < 1	2, 4 < 1 4 < 3	2, 3, 4 < 1	2 < 1	2, 4 < 1	2, 4 < 1

*Descriptive statistics (mean and SD) and F, p, and η^2^ values of the univariate analysis and post hoc tests of group differences.*

Globally, it emerged that the group with impaired performance in both verbal and non-verbal components of numeracy was similar to the one with only verbal numeracy difficulties, whereas children with typically developing numeracy skills and with a weakness involving only the non-verbal component of numeracy did not differ from each other in the language skills analyzed, and outperformed the other two groups.

## Discussion

The present study was aimed at providing a thorough examination of the relationship between language skills and early numeracy through a multilevel investigation of these skills in monolingual and bilingual children attending preschool. To our knowledge, this is the first study that has analyzed language-numeracy relationship in young pre-schoolers, with three levels of investigation being considered: group comparisons, predictors, and individual differences. A group of bilingual language minority children was included in order to offer a unique perspective into the role of linguistic competence in numerical development, and the profile analysis included in the study fosters the qualitative understanding of the strengths and weaknesses of the examined population, beyond and above the information derived from group mean scores.

The first aim of the present study was to investigate differences between bilingual and monolingual children in linguistic skills and early numeracy abilities. It was intended that this kind of analysis would help to ascertain the profile of the two populations in a wide set of tasks, in order to define patterns of strengths and weaknesses across multiple measures. As expected, there was a difference between groups in their linguistic profiles on all the linguistic tasks and this difference allowed us to look for a dissociation between linguistic and numerical skills. There was, however, an exception in the story sequencing task, for which the two groups did not differ. This task was the only one in the IDA battery with a non-verbal request and it was included because it was followed by a narrative task, which was not considered in the present study. When examining the significant differences, the smallest effect was found for phonological memory, a task involving non-word repetition. Despite involving non-familiar material for both monolinguals and bilinguals, the significant differences found for this task may depend on the fact that the stimuli were legal non-words; thus, their morphological structure adhered to Italian rules of word composition. It has been shown that, at least to a certain degree, bilinguals are sensitive to the morphological and distributional properties of the target L2 language (Bellocchi et al., 2016), and this is influenced by age of exposure. This may explain why in the non-word repetition task bilinguals lagged only marginally behind their monolingual peers.

The assessment of bilingual performance in L2 should be accompanied by an evaluation of L1 linguistic skills because highlighting weaknesses in L2 does not necessarily mean that the same pattern stands for L1 linguistic competencies. This is in line with International guidelines (American Speech-Language-Hearing Association [ASHA], 1985, 2004) on clinical assessment in multilingual contexts, and the lack of assessment of linguistic skills in L1 limits data interpretation as regards linguistic and working memory competence in the bilingual sample. However, considering the difficulties in assessing L1 competencies, particularly for language minority children, many studies have been aimed at studying linguistic and numerical competencies in L2, in order to gain information as to a typical bilingual developmental trajectory in L2 acquisition. Furthermore, in the case of numerical processing, it has been shown that bilingual children tend to be more proficient in solving numerical tasks when tested in their instructional language (Mondt et al., 2011), compared to those who were tested in their home language. In that case, the sole analysis of the L2 might be considered useful because it provides information about the instructional language; therefore, the identification of patterns of strengths and weaknesses in L2 may help to pinpoint potential risks and protective factors in the learning paths of bilingual children.

The pattern of difficulties in numerical skills was mixed. Bilinguals underperformed monolinguals in some numerical skills with a verbal component, such as semantic knowledge of digits, but they did not differ in pure non-verbal components such as quantity comparison. This pattern supports an independence of magnitude comparison from linguistic processing (Dehaene et al., 1999). Furthermore, they were similar to their monolingual peers in visuo-spatial memory. This task had very simple and mainly non-verbal instructions, and required a non-verbal answer. The similar performance observed in visuo-spatial memory that resulted in scores around the mean for both groups suggests that this important general cognitive non-verbal prerequisite was equally developed. Bilinguals’ gap in linguistic knowledge compared to monolinguals may explain their failing in the semantic knowledge of digits that involved the lexical retrieval of number names and grapheme-phoneme correspondences of Arabic digits. Despite this trend, bilinguals did not fail in counting ability, maybe because of the high frequency of the use of early counting sequences (i.e., 1–10) in everyday life or the high automaticity of sequencing number names. For an alternative explanation, the model of early counting competence with three basic components by Ferrara and Turner (1993) could be considered. They theorized (1) a verbal component involving knowledge of the conventional number-word sequence, (2) an action component involving knowledge of tagging behaviors in object-counting procedures, and (3) a contextual component involving knowledge of the basic goals and uses of counting. We can hypothesize that only the first component may be poorer in bilinguals compared to monolingual peers, and therefore the similar competence in the two remaining abilities would lead to a similar performance in the counting task. Finally, bilinguals underperformed monolinguals in some non-verbal components of number processing, such as number line and size seriation. This counterintuitive result might be explained by the characteristics of the verbal instructions given for these tasks, which were slightly more complex than the ones given for the other tasks, even though morpho-syntactic comprehension was not a significant predictor of these skills. An alternative hypothesis may be related to cultural characteristics of the stimuli such as the direction of the number line, as documented by studies with the SNARC effect in Arab populations (Dehaene et al., 1993). Further investigation is needed to increase knowledge on how these competencies develop in bilingual groups and, as discussed below, the lack of socio economic status (SES) measures limits the possibility to sustain definitive conclusions on the bilingual sample altogether.

The second aim was to analyze the pattern of linguistic predictors of number skills, in particular by investigating the two separate domains of verbal and non-verbal components of early numeracy. The analysis showed partially different patterns of predictors for the two groups considered. First, in both bilinguals and monolinguals, letter knowledge was a significant predictor of the verbal component of numeracy. The letter knowledge task required a grapheme-phoneme association as was, in some way, required by the semantic knowledge of the digits task included in the verbal component of numeracy. In other words, both tasks involved symbol processing. Furthermore, past studies showed medium to high correlations between tasks, such as naming speed, involving letters and digits (e.g., Bowey et al., 2005). These results suggest that symbol processing of letters and digits share a common underlying cognitive component independent from specific knowledge in one of the two domains. In monolinguals, there was also a marginally significant effect of vocabulary on the verbal component of numeracy, which is in line with past studies (e.g., Purpura and Ganley, 2014). Additionally, phonological awareness was, for the monolingual group, a significant predictor of the nonverbal component of numeracy skills. This result could be explained by the component of working memory involved in this task. In fact, working memory may serve as a link between phonological awareness and non-verbal early numeracy. In particular, the tasks of syllable blending and segmentation, beyond phonological processing, required an active component of working memory, namely the ability to manipulate phonological material before giving the target word. Working memory, as supported by past studies (Simmons et al., 2008; Zhang et al., 2014), is highly involved in early numeracy and was similarly involved in tasks included in the present study such as the mental number line where children were required to actively manipulate position in space depending on the target stimuli. Another mnemonic task, non-word repetition, had a passive memory component (rehearsal and repetition) that did not significantly predict numeracy skills.

The results from the bilingual group demonstrated, on the other hand, a substantial independence between linguistic skills and non-verbal numerical skills, because none of the linguistic measures were significant predictors of pure non-verbal numerical tasks. As far as pre-schoolers are concerned, only one study to date has analyzed cognitive (general intelligence, working memory) and linguistic precursors (phonological awareness, grammatical ability) to early numeracy in monolinguals and bilinguals (Kleemans et al., 2011). In line with results from the present study, the authors found that bilinguals had lower scores than first language learners in both linguistic and early numeracy tasks. Furthermore, correlation analysis showed that both phonological awareness and grammatical ability were needed for early numeracy development. Finally, no differences were detected in the pattern of correlations between language precursors and early numeracy for monolinguals versus bilinguals. The authors concluded that, in addition to cognitive factors, both phonological awareness and grammatical ability play equally important roles in the early numeracy of monolinguals and bilinguals. In Kleemans’ study children had a mean age of 6 years, all attended the last year of kindergarten, and thus were moderately exposed to literacy and number activities. Moreover, vocabulary, phonological memory and letter knowledge tasks were not included, although these linguistic measures are known to potentially influence number processing (Cirino, 2011; Purpura et al., 2011). In the present study, the reported pattern of relationships was observed in a very young sample of pre-schoolers (mean age 4.8 years) who had not yet been exposed to formal schooling, or to systematic activities on literacy and numerical skills. In fact, in the Italian school system activities on precursors of reading and math skills are mainly introduced by the second semester of the last year of preschool and these activities are mainly developed by class teachers. The National Indications (Istruzione, 2012) for the curriculum of preschool give general guidelines on the importance of promoting lexical and narrative skills, logical reasoning and spatio-temporal orientation. Thus, in the first two years of preschool, children are mainly exposed to playful didactic activities that have the broad aim of promoting the development of learning skills, but that do not include systematic and formal activities. Thus, the pattern of results described in the present study mainly refers to the spontaneous developmental trajectory of reading and math related skills. It is thus intriguing that letter knowledge plays a significant role from this early age. This observation reinforces previous studies (e.g., Caravolas et al., 2012) that proposed that the spontaneous ability of the child to acquire letter knowledge can be considered as a marker of his/her sensitivity to print exposure and of his/her ability to access some phonologic representations of speech. Thus, individual differences in letter knowledge in preschool years may be good predictors of literacy outcomes, and, based on the present study, also of numerical skills involving a verbal component.

Finally, the last aim of the study was to compare performance in verbal tasks for children with different profiles of early numeracy skills. Consequently, participants were further classified as having (1) no difficulties, (2) difficulties only in numerical tasks with a linguistic component, (3) difficulties only in non-verbal number tasks, and (4) difficulties in both components of early numeracy. First, an analysis of the frequency of monolinguals and bilinguals across the four categories showed a similar distribution, suggesting that numerical weaknesses were equally distributed in the two subsamples. This is in line with Kleemans et al. (2011), who suggested that a gap in numerical skills in bilingual children, also found in the present study in some numerical skill group comparisons, might be better accounted for as an educational delay rather than as a clinical impairment. In fact, analysis of differences in early numerical skills showed a small to medium (Cohen, 1988) gap between bilinguals and monolinguals, and their mean scores fell within the typical range (see scaled scores). This result, together with the additional information offered by the profile analysis, delineates a group of children with mild difficulties in precursors of mathematical abilities. This outcome suggests the potential role that an early targeted intervention could play, together with good practices in the classroom, in reducing this gap (e.g., Fuchs et al., 2005; Klibanoff et al., 2006). These measures would be more effective if the specific profile of difficulties showed by each child were considered. Profile analysis also showed that children with a selective weakness in the non-verbal component of numeracy had mostly adequate verbal skills. This represents a complementary perspective on the relative independence of linguistic and numeracy domains, at least for those skills related to magnitude comparison and for what is referred to as the ANS. An interesting result is the profile of language skills that emerged for children with a poor verbal component of numeracy: they showed good vocabulary skills and particularly lower scores in phonological tasks (phonological memory and awareness) and in letter knowledge. This result is in line with past studies that showed the importance of these variables in influencing some key components of mathematical abilities (e.g., Simmons and Singleton, 2008), and adds information regarding the influential and early role of letter knowledge.

This study had some limitations, specifically the paucity of information regarding participants’ SES and the level of L1 proficiency in the bilingual group, which may limit the generalizability of the results and makes it difficult to draw any firm conclusions regarding the bilingual sample as a whole. These variables need to be better accounted for in future cross-group comparisons. However, the main aim of the present study was to evaluate predictors and patterns of strengths and weaknesses in linguistic and numerical skills, rather than absolute levels of performance and group differences between bilinguals and monolinguals. Furthermore, studies investigating the effects of SES on early mathematics found that preschoolers from low socio-economic backgrounds have difficulties in most early mathematical skills, such as counting, comparing magnitudes and performing simple calculations (e.g., Jordan et al., 2006). The pattern of results we found, with only some components of early mathematical abilities being affected, suggests a main role for language-related underexposure, rather than SES, in explaining our results. We suggest that the selection criteria adopted, together with the minor predictive role of SES in Italy (OCSE-Programme for International Student Assessment [PISA], 2006), contribute to substantially minimize the role of SES in explaining the pattern of results discussed here.

Finally, it is worth underlining the importance of children’s capability to understand instructions when the relationship between their linguistic and numerical skills is being analyzed. Previous studies (Vukovic and Lesaux, 2013) found that in 6- to 9-year old monolingual and bilingual children, significant correlations emerged between language and skills in data analysis and geometry, whereas there were no correlations with arithmetic or algebra skills. The authors suggested that linguistic abilities are helpful in understanding meaning, but they are not essential to perform well in tasks requiring the use and elaboration of Arabic symbols and quantities. In this study, we tried to minimize verbal instructions of tasks; furthermore, we used examples and we checked carefully that all children understood what they were requested to do in each task. However, it is plausible that L2 instructions represented a stronger challenge for bilingual than monolingual participants. Nevertheless, in the present study no significant relationships between morpho-syntactic comprehension and early numerical skills were found, suggesting that the ability to perform in numerical tasks was not primarily related to the ability to comprehend L2 sentences.

## Conclusion

To conclude, this is the first study that investigates cognitive and linguistic underpinnings of early numeracy in preschool bilingual and monolingual children at different levels of analysis; the results suggest that only some specific components of language competence may predict specific numerical processing. However, having a good level of linguistic proficiency may not be a necessary condition in order to develop adequate abstract representation of numbers, as supported by the absence of a relationship between language and numeracy in bilingual children with overall adequate number competencies and weak linguistic skills (as shown when discussing the first aim). Linguistic weaknesses may lead to poorer performance with numeracy but not to impaired numerical abilities. In fact, the scaled scores for all the number and number-related tasks administered were in the average range for the children’s age, and there were no differences in the proportion of monolinguals and bilinguals showing significant weaknesses in numeracy skills.

The lack of L1 measures of proficiency requires further evaluation in future studies, in order to better disentangle the role of language skills in specific numerical processing. However, in the present study children were tested in their instructional language and, although they are still in preschool years, it is reasonable to hypothesize that they have acquired basic number abilities in the school setting, albeit in the absence of a formal instruction. Therefore, the results from the present study allow us to assume that there is a relative independence of linguistic and numerical processing in bilingual language minority children with a gap in linguistic development, compared to their monolingual peers. In order to investigate the relationship between linguistic and numerical skills in depth we included a sample of bilingual children because of their particular differential language exposition. Further investigations might focus on clinical populations of children with language impairments, who are known to have specific weaknesses in the phonological and lexical domains.

In summary, these findings offer new insight both for clinical and educational settings, suggesting the importance of defining proper assessment of strengths and weaknesses, and targeting intervention to specific domains in minority bilingual children. Furthermore, from a theoretical perspective, the present study suggests that at the very beginning of literacy and numerical development the two domains are relatively independent.

## Author Contributions

PB, VT, and GM contributed to the conception and design of the work; VT, LB contributed substantially to data acquisition. All authors were involved in analysis and interpretation of data for the work. PB, VT, and LB drafted the work and GM revised it critically. All authors finally approved the present version of the manuscript and agree on all aspects of the work.

## Conflict of Interest Statement

The authors declare that the research was conducted in the absence of any commercial or financial relationships that could be construed as a potential conflict of interest.
